# Fecal Microbiota Transplantation Could Improve Chronic Diarrhea in Cynomolgus Monkey by Alleviating Inflammation and Modulating Gut Microbiota

**DOI:** 10.3390/biomedicines10123016

**Published:** 2022-11-23

**Authors:** Puyuan Tian, Jiangmei Gao, Lifeng Liang, Bota Cui, Qiming Hu, Wenfeng Zhou, Bihai Li, Yiyan Liu, Tingtao Chen, Junhua Rao, Hong Wei

**Affiliations:** 1 Institute of Precision Medicine, The First Affiliated Hospital, Sun Yat-sen University, Guangzhou 510080, China; 2 Guangdong Key Laboratory of Animal Conservation and Resource Utilization, Guangdong Public Laboratory of Wild Animal Conservation and Utilization, Institute of Zoology, Guangdong Academy of Sciences, Guangzhou 510275, China; 3 Medical Center for Digestive Diseases, The Second Affiliated Hospital of Nanjing Medical University, Nanjing 210011, China; 4 Guangzhou Xiangguan Biotechnology Co., Ltd., Guangzhou 510900, China; 5 National Engineering Research Center for Bioengineering Drugs and the Technologies, Institute of Translational Medicine, Nanchang University, Nanchang 330036, China

**Keywords:** chronic diarrhea, cynomolgus monkey (*Macaca fascicularis*), fecal microbiota transplantation (FMT), gut bacteria, fungi, Archaea

## Abstract

Chronic diarrhea is associated with enteric dysbiosis and provokes the overuse of antibiotics. Fecal microbiota transplantation (FMT) is a promising therapy, but it shows discrepant clinical efficacy. Bacterial colonization in recipients has been studied, although little is known about the role of gut fungi and Archaea after FMT. In this study, we evaluated the efficacy of human-derived FMT on spontaneous chronic diarrhea cynomolgus monkeys and revealed the effector mechanisms. We demonstrated that FMT can mitigate the appearance of diarrheal symptoms and inhibit the increase in interleukin-6, interleukin-8, interleukin-1β, and interferon-γ and the decrease in interleukin-10 in serum. We confirmed that FMT restored the disturbance of gut bacteria by reducing the relative abundances of potential pathogens, including *Cloacibacillus porcorum, Desulfovibrio desulfuricans, Erysipelotrichaceae bacterium* 5_2_54FAA, and *Erysipelotrichaceae bacterium* 21_3, and increasing the levels of *Lactobacillus fermentum* and *Lactobacillus ruminis* CAG_367 in diarrheal monkeys. The metabolic pathways of healthy and FMT monkeys’ gut bacteria were enriched in amino acid metabolism, carbohydrate metabolism, and lipid metabolism, while the metabolic pathways of pre-FMT monkeys’ gut bacteria were enriched in antibiotic production. Moreover, a higher Ascomycota/Basidiomycota ratio, higher *Aspergillus* levels, and lower *Trichosporon asahii* abundance were present in intestinal fungi after FMT. Although the abundance of the Archaea *Methanosphaera stastmanae* did not change significantly, it was inversely correlated with the anti-inflammatory factor IL-4 after FMT. These results support the further development and application of FMT for chronic diarrhea.

## 1. Introduction

Chronic diarrhea is a common and complex functional bowel disorder that includes two mutually exclusive subgroups: irritable bowel syndrome diarrhea (IBS-D) and functional diarrhea (FD) [1]. With an estimated prevalence of 3–20% worldwide [2,3], it has a significant negative impact on health-related quality of life, but the underlying pathophysiology remains unclear. Antibiotics can effectively relieve acute and chronic diarrhea by acting on gut microbiota [4]. However, antibiotic abuse destroys microbe diversity and homeostasis in the gastrointestinal tract and induces the rapid spread of multi-resistant bacteria, and approximately 5–35% of patients develop antibiotic-associated diarrhea (AAD) [5] further complicates the treatment of chronic diarrhea and has long-term negative consequences on human healthcare [6]. Therefore, understanding the pathogenesis and exploring alternative treatments for chronic diarrhea are urgently needed [7].

Fecal microbiota transplantation (FMT), which is the instillation of fecal contents from healthy donors into recipient patients for normalizing or restoring gut microbiota, is considered a promising therapeutic pathway for intestinal microbiota related diseases [8,9]. FMT has been successfully used as a highly effective therapy for recurrent *Clostridioides difficile* infections (CDI) [10,11], which account for approximately one-third of AAD cases [5]. This success has sparked interest in extending its application to other diseases, such as inflammatory bowel disease (IBD), IBS, autoimmune disorders and metabolic syndrome [12,13]. In contrast to the 85–90% treatment success for CDI, randomized clinical trials in Norway (with 90 patients) [14,15] and in the United States (with 48 patients) [16] showed FMT application in IBS could be both effective and ineffective in relieving clinical symptoms because of the complex disease etiology and unclear microbial target [17]. This heterogeneity highlights the fact that more extensive mechanistic studies are required to elucidate the roles of the gut microbiota and to design specialized therapy for patients [18].

Preclinical animal models are commonly used tools for studying disease pathogenesis or new therapies, and chemically induced small-animal models, especially rodent models, are the most popular. The translatability of these models to human diseases is poor but may be improved by using animals that are closely genetically and pathologically related to humans [19]. Owing to the genetic and physiological similarities between non-human primates (NHPs) and humans, NHPs are suitable as model organisms for fundamental research. Importantly, captive cynomolgus monkeys (*Macaca fascicularis*) have been reported to undergo indigenous microbe loss, and the primary bacterial genera in their gut are *Prevotella* and *Bacteroides*, which have genes more similar to the gut bacterial genes in humans (39.49%) compared with those in pigs (25.45%) and mice (0.6%) [19]. Furthermore, spontaneous diarrheal models display many of the clinical and pathological features of human diseases. In summary, captive spontaneous diarrheal monkeys may serve as appropriate models for understanding human chronic-diarrhea-susceptibility factors and validating microbiota interventions [7,20,21]. 

Previously, 16S rRNA sequencing was used to assess the restoration of bacterial microbiota compositions as a descriptive method for FMT treatment outcomes [22]. However, this assessment is imperfect, providing little information on bacterial function, and parsing the “transmit” system incompletely [11]. Fungi (the “mycobiome”) and Archaea are both stable components of the microbiota and contribute to normal human physiology [23,24]. IBD and colorectal cancer (CRC) patients have both been shown to have a decreased Ascomycota/Basidiomycota ratio and higher levels of methanogenic Archaea than healthy controls [25,26,27]. In addition, the gut-resident mycobiome and Archaea have mutualistic and antagonistic relationships with the gut bacteria, working together to shape host immunity [17]. In CRC progression, trans-kingdom interactions were reported to occur between enriched fungi (*Aspergillus rambellii*) and bacteria (*Fusobacterium nucleatum*). CRC-associated Archaea (halophiles) were positively associated with oncogenic *Bacteroides fragilis* and inversely associated with probiotic *Clostridium* [28]. Moreover, recent studies have reported that FMT involves the transfer of fungi, which can affect treatment outcomes in *C. difficile* infections, ulcerative colitis (UC), and Crohn’s disease (CD) [17]. Whether or not donor-derived mycobiota and Archaea can colonize recipient hosts, and the identity of the causal relationships between gut fungal, Archaeal and FMT treatment outcomes in chronic diarrhea are unknown.

Consequently, in this study, we evaluated the therapeutic effect of human-derived FMT on chronic diarrhea by using spontaneous monkey models. The immune responses in the hosts were assessed by measuring changes in serum cytokines. Subsequently, fecal metagenomes were used to compare the gut microbial compositions and functional pathways. Simultaneously, gastrointestinal fungi and Archaea were profiled by amplicon sequencing. These basic data support the use of FMT as an alternative therapy that can be used to alleviate chronic diarrhea and serve as a valuable reference and resource for mechanism research using the monkey as a model. 

## 2. Materials and Methods

### 2.1. Study Subjects and Treatment Outcomes 

The captive monkeys in this study were provided by Guangzhou Xiangguan Biotechnology Co., Ltd., and were housed in standard conditions (temperature 16–26 °C, humidity 40–70%) in Guangzhou (Guangdong, China). The monkeys had recurrent diarrhea, and after repeated antibiotic treatment they then developed chronic diarrhea. Three monkeys with chronic diarrhea and four healthy monkey controls were included. All seven monkeys were maintained in similar environments and were given free access to food and water. Neither the healthy monkeys nor the chronic diarrhea monkeys received antibiotic treatment for 1 month prior to FMT intervention.

The fecal bacteria suspension from a healthy donor was from the Chinese fmt-Bank (Nanjing, Jiangsu, China). The healthy donor was a 26-year-old female, and the concentration of the fecal bacterial suspension was 10^7^ CFU/5 mL. Chronic diarrhea monkeys received fecal suspension four times via nasogastric gavage (every other day). Three fresh feces samples from chronic diarrhea monkeys before FMT (Day-0), nine fresh feces samples from chronic diarrhea monkeys after FMT (three from Day-2, Day-4, and Day-8), and four fresh feces samples from healthy monkeys were collected for analyses of bacteria, fungi, and Archaea in the microbiome. Blood samples (3–5 mL) were collected from all seven monkeys concurrent with the feces collection for serum inflammatory cytokines analyses (Supplementary Table S1 and Figure S1A). No monkeys were injured during the intervention or the collection of feces and blood, and our study did not affect the health and welfare of the monkeys. This study was approved by the Ethics Committee of the Institute of Zoology, Guangdong Academy of Sciences (No. GIZ20220105).

### 2.2. ELISA

The levels of interleukin-4 (IL-4), interleukin-6 (IL-6), interleukin-8 (IL-8), interleukin-10 (IL-10), interleukin-1β (IL-1β), and interferon-γ (IFN-γ) in serum were measured using ELISA kits (Meimian Industry Co., Ltd., Yancheng, Jiangsu, China) following the manufacturer’s instructions. In detail, standard and sample diluent were added; the mixture was incubated for 30 min at 37 °C, and then washed five times. HRP-conjugate reagent was added, and the mixture incubated for 30 min at 37 °C, then washed five times. We added chromogen solutions A and B, incubated the mixture for 10 min at 37 °C, and added stop solution. The absorbance was read at 450 nm within 15 min and the concentrations were calculated. 

### 2.3. Metagenomics and Functional Prediction Analyses 

DNA in the monkey fecal samples was extracted using a DNA extraction kit (Tiangen Biotech Co., Ltd., Beijing, China) according to the protocol. The extracted DNA was quantified in a NanoDrop spectrophotometer. After fragmentation and index-containing adaptor ligation, samples were paired-end sequenced on an Illumina NovaSeq 6000 platform (2 × 150 bp, Novogene Co., Ltd., Beijing, China). Clean data were obtained by filtering adapters and low-quality reads from the raw data using Trimmomatic [29], and potential cynomolgus monkey sequences were removed by Bowtie2 (V2.4.2) [30] based on the Macaca fascicularis reference genome (assembly Macaca_fascicularis_5.0 (RefSeq GCF_000364345.1)). 

Metagenome assembly was performed using SOAPdenovo (V2.04) [31] with the option “-d 1-M 3”. Then, ambiguous bases were removed from the assembled scaffolds (this divided one scaffold into multiple ones), and scaffolds with lengths less than 500 bp were discarded. Finally, a series of k-mer values (from 31 to 59) were tested, and the values with the longest N50 value were chosen for the remaining scaffolds [32]. We used MetaGeneMark (http://exon.gatech.edu/GeneMark/metagenome/Prediction, V2.10, 4 March 2015) to predict ORFs in scaffolds without ambiguous bases [33]. Dereplication of the microbial gene catalog was performed with CD-HIT (V4.8.1), using a global identity threshold of 95% and coverage of 90% (-c 0.95 -aS 0.9) [34]. SOAPalign (V2.21) [35] was used to align paired-end clean reads against the nonredundant microbial gene catalog with parameters “-M 4 -r 2 -m 100 -× 1000”. We calculated the relative abundance of phyla, genera, species, and KOs from the relative abundance of their respective genes using previously published methods [32].

Functional annotation according to the Kyoto Encyclopedia of Genes and Genomes (KEGG) using BLAST (V2.2.28+, http://blast.ncbi.nlm.nih.gov/Blast.cgi, 13 April 2014) based on the “GENES” database was used to visualize metabolism-associated functions. A similar pipeline was used for the Comprehensive Antibiotic Resistance Database (CARD) (V3.1.3) [36].

### 2.4. High-Throughput Amplicon Sequencing 

For fungi, specific barcoded primers F (5′-GGAAGTAAAAGTCGTAACAAGG-3′) and R (5′-GCTGCGTTCTTCATCGATGC-3′) were used to amplify the ITS1-2 region of 16S rRNA genes in each sample, according to methods described by Luan et al. [37]. For Archaea, the primer pair 1106F (5′-TTWAGTCAGGCAACGAGC-3′) and 1378R (5′-TGTGCAAGGAGCAGGGAC-3′) were used with a pair of 8-bp forward and reverse barcode sequences to amplify the Archaea region of the 16S rRNA genes [38]. The 1106F/1378R primer set mainly targets methanogenic Archaeal 16S rRNA genes but can still detect non-methanogenic clades due to non-specificity [39].

### 2.5. Quantification and Statistical Analysis

For the comparison of two groups, the Student *t*-test was selected after testing for normality of data and homogeneity of variance. For the comparison of three or more groups of non-normal measurements, the Kruskal-Wallis test with Benjanini-Hochberg (BH) correction was employed for statistical analysis. Differences were considered significant at *p* < 0.05. Spearman correlation analysis was used for correlations between microbial taxa abundances, metagenomic function abundances, and phenotype data. The significance cutoff for correlation was set at a BH-adjusted *q* < 0.05. All plotting was performed using R packages “ggpubr” (V0.4.0), “Com-plexHeatmap” (V2.13.1), and “mixOmics” (V6.1.1). 

## 3. Results

### 3.1. FMT Ameliorates Diarrhea in Captive Monkeys

**Figure 1:**
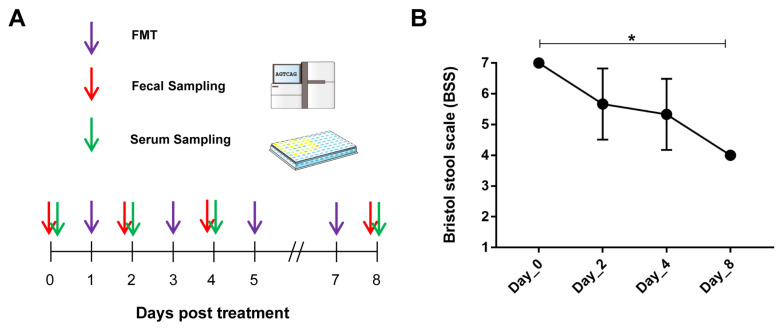
Efficacy of human-derived FMT ameliorates diarrhea in captive monkeys. (**A**) Design of FMT trial in monkeys with diarrhea. Detailed description is provided in the Materials and Methods section. (**B**) The diarrheal score was represented by the Bristol stool scale (BSS) for the fecal samples collected from chronic diarrhea monkeys before (Day-0) and after (Day-2, Day-4, Day-8) FMT intervention (*n* = 3 for all timepoints). *p*-values were determined using Dunnet’s multiple comparisons test. * *p* < 0.01.

To investigate the effect of FMT from a healthy human donor on diarrheal monkeys, we administered three diarrheic monkeys via nasogastric gavage, regardless of the presence of diarrhea, with 10^7^ CFU/5 mL fecal bacteria suspension four times (every other day, Figure 1A). We then closely observed these monkeys for 2 weeks and found no signs of abnormal behavior or acute illness. Examination of the collected samples showed that FMT reduced the incidence of diarrhea in the recipient monkeys (Figure S1A). Analysis of the visual indicators (Bristol stool scale, BSS, Supplementary Table S2) showed that there was significantly lower fecal liquidity on Day-8 after FMT intervention (7 vs. 4, *p* < 0.05), indicating symptoms of chronic diarrhea had been partially relieved (Figure 1B). Next, we performed an inflammation assessment of serum and microbial analysis of feces collected from healthy monkeys (HC) and diarrheal monkeys just before (Day-0) and on Day-2, Day-4, and Day-8 after FMT intervention.

### 3.2. FMT Mitigates Serum Inflammation Response in Chronic Diarrhea Monkeys 

**Figure 2:**
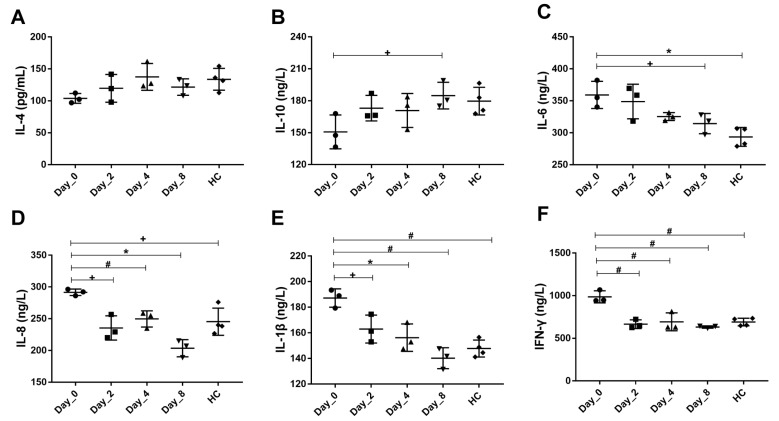
Serum inflammatory cytokine levels of diarrheic monkeys following FMT treatment. (**A**–**F**) Concentrations of interleukin-4 (IL-4) (**A**), interleukin-10 (IL-10) (**B**), interleukin-6 (IL-6) (**C**), interleukin-8 (IL-8) (**D**), interleukin-1β (IL-1β) (**E**), and interferon-γ (IFN-γ) (**F**). HC, healthy monkeys (*n* = 4); Day-0 (D0), chronic diarrhea monkeys before FMT intervention (*n* = 3); Day-2 (D2), Day-4 (D4), Day-8 (D8), chronic diarrhea monkeys after FMT intervention (*n* = 3 for all timepoints). *p*-values were according to one-way ANOVA followed by Dunnet’s multiple comparisons test. + *p* < 0.05, * *p* < 0.01, # *p* < 0.001.

To further explore the effect of FMT intervention on the immune system, serum IL-4, IL-10, IL-6, IL-8, IL-1β, and IFN-γ levels were measured. Compared with healthy monkeys, chronic diarrhea monkeys had lower concentrations of IL-4 and IL-10 and significantly higher concentrations of IL-6, IL-8, IL-1β, and IFN-γ (Figure 2A–F). However, FMT intervention effectively enhanced the levels of the anti-inflammatory cytokine IL-10 (D8 vs. D0, 184.85 vs. 150.73, *p* < 0.05), and inhibited the levels of the pro-inflammatory cytokines IL-6 (D8 vs. D0, 314.425 vs. 359.28, *p* < 0.05), IL-8 (D8 vs. D0, 203.57 vs. 291.63, *p* < 0.01), IL-1β (D8 vs. D0, 140.2 vs. 187.22, *p* < 0.001), and IFN-γ (D8 vs. D0, 632.29 vs. 985.97, *p* < 0.001, Figure 2C–F). 

### 3.3. FMT Alters the Intestinal Bacteria Composition of Chronic Diarrhea Monkeys

To determine temporal changes in the monkey gut bacteria in response to FMT treatment, metagenomic analysis was performed at three time-points after FMT. Firmicutes presented at the highest proportion in monkey guts, and the population expanded in chronic diarrhea monkeys returned to levels comparable to healthy monkeys after FMT intervention (D0 vs. D8 vs. HC, 65.26% vs. 69.41% vs. 70.39%, Figure S1B). The guts of healthy monkeys were dominated by *Lactobacillus,* and FMT monkeys showed an increased abundance of *Lactobacillus* compared with pre-FMT monkeys (HC vs. D0 vs. D8, 31.91% vs. 6.90% vs. 13.08%, Figure S1C). 

**Figure 3:**
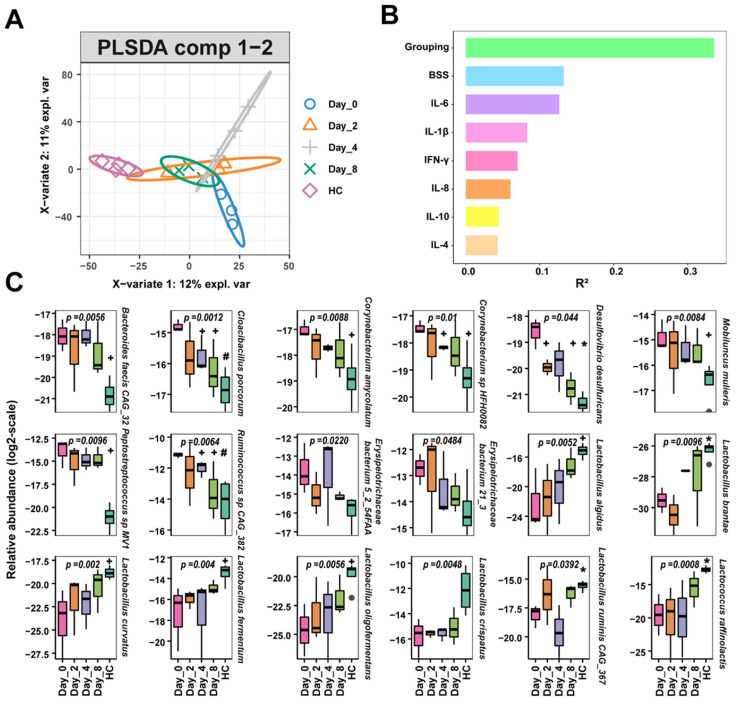
Fecal bacteria changes in diarrheic monkeys following FMT treatment. (**A**) PLS-DA plot of species-level relative abundance profile. (**B**) The microbial variations explained by phenotypes (PERMANOVA). (**C**) Comparison of main bacterial species of all 5 groups. HC, healthy monkeys (*n* = 4); Day-0 (D0), chronic diarrhea monkeys before FMT intervention (*n* = 3); Day-2 (D2), Day-4 (D4), Day-8 (D8), chronic diarrhea monkeys after FMT intervention (*n* = 3 for all timepoints); Grouping, HC, Day-0, Day-2, Day-4, Day-8; BSS, Bristol stool scale. Compared with Day-0, + *p* < 0.05, * *p* < 0.01, # *p* < 0.001. *p*-value represented the difference of log2 ratio-transformed relative abundance on either Day-0 or in HC compared with other groups.

Partial least squares discriminant analysis (PLS-DA) revealed distinct clusters of intestinal species in healthy monkeys, pre-FMT monkeys, and FMT monkeys. Additionally, similar species compositions were seen at different timepoints during the FMT intervention, suggesting that a new microbiota homeostasis structure was formed after FMT (Figure 3A). When analyzed, grouping, BSS and IL-6 levels were linked to the top three variations in the microbial species compositions (PERMANOVA, R^2^ = 0.3355, 0.1324 and 0.1265 respectively, Figure 3B), whereas serum inflammatory factors (i.e., IL-4, IL-10, IL-8, IL-1β and IFN-γ) did not impact microbial species composition significantly (all R^2^ < 0.1, Supplementary Table S3). Several *Lactobacillus* and *Lactococcus* species (log2 ratio-transformed relative abundance) such as *Lactobacillus fermentum*, *Lactobacillus ruminis* CAG_367 and *Lactococcus raffinolactis*, were enriched in healthy monkeys (HC, *p* = 0.004, 0.0392 and 0.0008 respectively) and showed a tendency to increase after FMT intervention (Figure 3C). Several potential pathogens (log2 ratio-transformed relative abundance), including *Cloacibacillus porcorum* and *Desulfovibrio desulfuricans*, were enriched in diarrheal monkeys (D0, *p* = 0.0012 and 0.044, respectively) and showed a tendency to decrease after FMT intervention (Figure 3C).

### 3.4. FMT Changes in Gut Bacteria Compositions Associated with Functional Changes 

**Figure 4:**
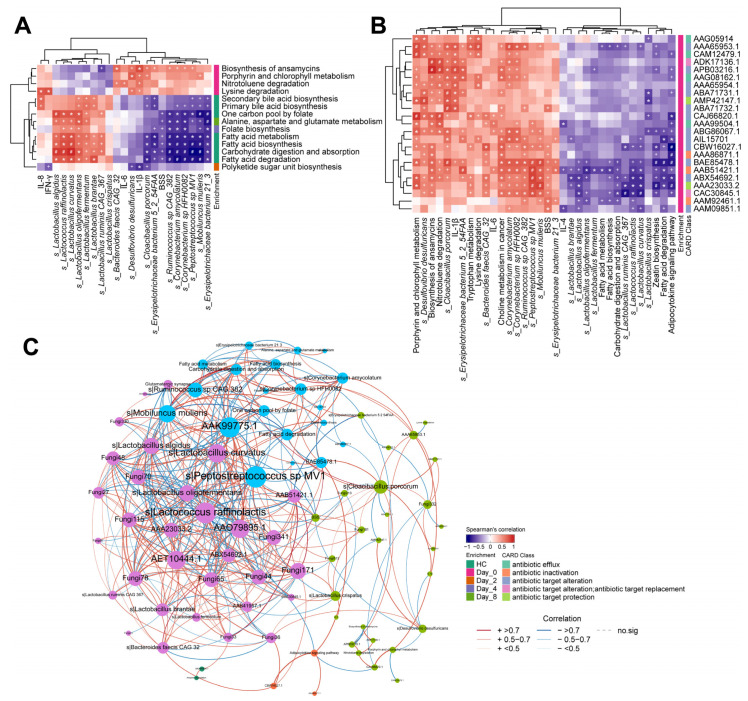
Discriminating bacteria functions associated with phenotype and correlation analyses of altered bacteria species. (**A**) Correlation between the KEGG functional pathways, phenotypes, and microbials. Color indicates significantly positive association (red), significantly negative association (blue), or insignificant association (white). (**B**) Correlation (Spearman) between KEGG functional pathways, CARD protein names, and microbial species. Color indicates significantly positive association (red), significantly negative association (blue), or insignificant association (white). + *p* < 0.05, * *p* < 0.01, # *p* < 0.001. (**C**) Feature-to-feature association networks. Each connection represents a strong (Spearman’s correlation coefficient, R^2^ > 0.5) and significant (*q* < 0.05) correlation. Edges were weighted according to the correlation coefficient, and label size was weighted according to the number of networks. The significance cutoff for correlation was set at a BH-adjusted *p* < 0.05.

We next identified the functional modules associated with the clinical phenotypes and assessed the associations with species among these modules. The results indicated that seven KEGG pathways related to amino acid metabolism, carbohydrate metabolism and lipid metabolism enriched in healthy monkeys and FMT monkeys were negatively correlated with BSS (*p* < 0.05). In addition, four KEGG pathways enriched in pre-FMT monkeys were positively correlated with IL-6, IL-8, IL-1β and IFN-γ (*p* < 0.05). We further assessed the associations between these microbial KEGG pathways and species alterations after FMT intervention. The seven KEGG pathways enriched in healthy monkeys and FMT monkeys were found to be positively correlated to different degrees with *Lactobacillus* species (*p* < 0.05), including *L. fermentum* (6) and *L. ruminis* CAG_367 (4). Additionally, different degrees of negative correlation were also found between potential pathogens, including *C. porcorum* (4), *Erysipelotrichaceae bacterium* 5_2_54FAA (4), *Erysipelotrichaceae bacterium* 21_3 (7), and the seven KEGG pathways enriched in healthy monkeys and FMT monkeys. Moreover, *L. ruminis* CAG_367 had a negative correlation with the enriched biosynthesis of ansamycins in pre-FMT monkeys. *D. desulfuricans*, *C. porcorum*, and *Erysipelotrichaceae bacterium* 5_2_54FAA had positive correlations with the enriched biosynthesis of ansamycins in pre-FMT monkeys (*p* < 0.05, Figure 4A). 

We also identified the microbial genes encoding antibiotic resistance factors by sequence classification with reference to the CARD database. Notably, all five CARD classes (antibiotic efflux, antibiotic inactivation, antibiotic target alteration, antibiotic target replacement and antibiotic target protection) were enriched in pre-FMT monkeys. Moreover, the five CARD classes were positively associated with diarrhea-related bacteria (*D. desulfuricans*, *C. porcorum*, *Erysipelotrichaceae bacterium* 5_2_54FAA, *Erysipelotrichaceae bacterium* 21_3). Additionally, diarrhea-improvement bacteria (*L. fermentum*, *L. ruminis* CAG_367) were negatively associated with the five CARD classes (*p* < 0.05, Figure 4B). The complete correlation networks between the microbiota, functional pathways, and phenotypes mentioned above (Figure 4A,B) are presented in Figure 4C (*q* < 0.05).

### 3.5. FMT Alters Gut Fungi of Chronic Diarrhea Monkeys in Relation to Treatment Responses 

**Figure 5:**
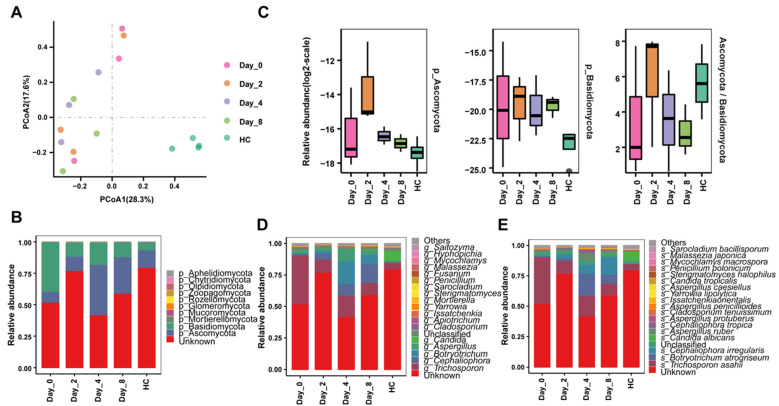
Fecal fungi changes in diarrheic monkeys following FMT treatment. (**A**) PCoA plot based on Bray-Curtis distance of ASV abundance. (**B**) Phyla-level taxonomic profiles of gut fungi from five groups. (**C**) Relative abundances of Ascomycota and Basidiomycota and ratio of Ascomycota to Basidiomycota in five groups. (**D**) Genus-level taxonomic profiles of the gut fungi from five groups. (**E**) Species-level taxonomic profiles of gut fungi from five groups. HC, healthy monkeys (*n* = 4); Day-0 (D0), chronic diarrhea monkeys before FMT intervention (*n* = 3); Day-2 (D2), Day-4 (D4), Day-8 (D8), chronic diarrhea monkeys after FMT intervention (*n* = 3 for each timepoint).

To investigate whether FMT leads to colonization by donor-derived fungi and the association between this decolonization and treatment efficacy, we executed ITS amplicon sequencing of fecal samples at three timepoints after FMT. A PCoA plot based on the ASV-level relative abundance profile showed that axis 1 (PCoA1) explained 28.3% of the variability and axis 2 (PCoA2) explained 17.6% of the variability. The fungal compositions of healthy monkeys varied from those of pre-FMT monkeys and FMT monkeys (R^2^ = 0.4166, *q* = 0.0099, Figure 5A). At the phyla level, Ascomycota, Basidiomycota, and an unknown phylum together accounted for up to 99.5% of bacterial abundances in healthy monkeys, pre-FMT monkeys, and FMT monkeys (Figure 5B). Previous work revealed that IBD patients had dysbiotic mycobiota and a lower Ascomycota/Basidiomycota ratio, which prompted us to study the Ascomycota/Basidiomycota ratio in our cohort. We observed a trend toward a higher Ascomycota/Basidiomycota ratio in feces from FMT monkeys compared to those of pre-FMT monkeys (D2 vs. D4 vs. D0, 5.90 vs. 3.54 vs. 3.46, Figure 5C).However, these differences did not reach statistical significance, possibly due to the limited sample sizes. At the genera level, pre-FMT monkeys showed an increased relative abundance of *Trichosporon* compared with healthy monkeys, and FMT intervention partly reversed this trend (HC vs. D0 vs. D8, 5.32% vs. 38.64% vs. 9.82%). The relative abundance of *Aspergillus* in FMT monkeys was greater than that in pre-FMT monkeys (D4 vs. D0, 9.56% vs. 1.30%, Figure 5D). *Trichosporon asahii* had a lower abundance in chronic diarrhea monkeys after FMT intervention, and the levels were more similar to those in healthy monkeys (D0 vs. D8 vs. HC, 38.64% vs. 9.82% vs. 5.32%, Figure 5E). 

### 3.6. FMT Alters the Gut Archaea of Chronic Diarrhea Monkeys in Relation to Treatment Responses

**Figure 6:**
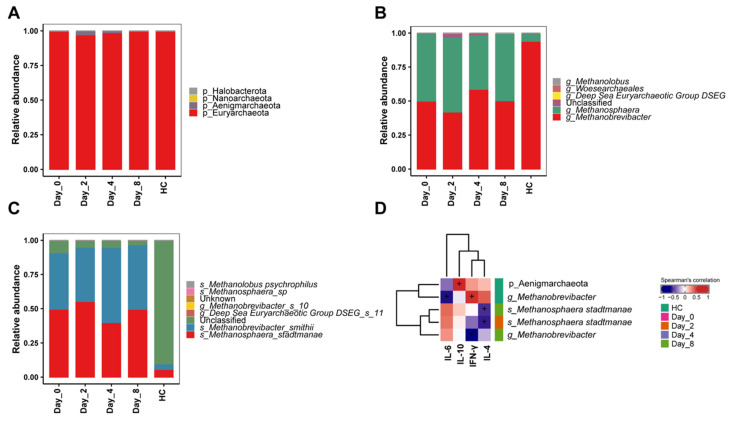
Fecal Archaea changes in diarrheic monkeys following FMT treatment. (**A**) Phyla-level taxonomic profiles of gut Archaea from five groups. (**B**) Genus-level taxonomic profiles of gut Archaea from five groups. (**C**) Species-level taxonomic profiles of gut Archaea from five groups. (**D**) Correlation (Spearman) between the ASVs and phenotypes. Color indicates significant positive association (red), significant negative association (blue), or insignificant association (white). HC, healthy monkeys (*n* = 4); Day-0 (D0), chronic diarrhea monkeys before FMT intervention (*n* = 3); Day-2 (D2), Day-4 (D4), Day-8 (D8), chronic diarrhea monkeys after FMT intervention (*n* = 3 for each timepoint). + *p* < 0.05.

To explore the dynamics of the intestinal Archaea in monkeys undergoing FMT, we analyzed the fecal Archaea at three timepoints after FMT by amplicon sequencing. Euryarchaeota constituted the most dominant phyla in monkey guts, with more than 97% relative abundance (Figure 6A). A deeper analysis of the Archaea taxonomic composition revealed *Methanobrevibacter* and *Methanosphaera* were the most abundant genera (Figure 6B). Within *Methanobrevibacter* and *Methanosphaera*, *Methanobrevibacter smithii* and *Methanosphaera stadtmanae* were the most abundant and prevalent species (Figure 6C). Nevertheless, quite distinct enterotypes were observed. Healthy monkeys were characterized by the predominance of *Methanobrevibacter* (93.95%), while chronic diarrhea monkeys both before and after FMT had uniform distributions of *Methanobrevibacter* (D0 vs. D8, 49.99% vs. 50.18%) and *Methanosphaera* (D0 vs. D8, 49.59% vs. 49.49%). We next analyzed the correlations between Archaeal compositions (at the ASV level) and phenotypic factors. Although FMT did not significantly alter *M. stadtmanae* abundance, negative association was observed between *M. stadtmanae* and IL-4 in FMT monkeys (*p* < 0.05, Figure 6D).

## 4. Discussion

Chronic diarrhea is a common functional bowel disease and includes the two subtypes IBS-D and FD, and gut microbiota disturbance has been considered a possible cause [3]. The outcomes of several pilot and larger-scale clinical trials confirmed the validity of applying FMT in the treatment of diseases with complex etiology such as IBD and IBS, but significant challenges have also presented themselves because of the complex disease etiology and the lack of a clear microbial target [40]. We used chronic diarrhea monkey models to evaluate the effectiveness of human-derived FMT with a particular focus on the mechanisms. Our results demonstrated that human-derived FMT had significant relieving effects on chronic diarrhea in monkeys via the regulation of immune responses and the modulation of intestinal microbiota disturbances.

BSS, which combines reference pictures with standardized descriptions of stool form, is a widely used, reliable, and validated assessment method for chronic diarrhea and is one of the outcome measures recommended by the Federal Drug Administration (FDA) for use in IBS-D clinical trials [41]. We found significantly lower BSS on Day-8 after FMT intervention (Figure 1B). Studies have pointed out that the increase in pro-inflammatory cytokines (IL-6, IL-8, TNF-α, and IFN-γ) and decrease in the anti-inflammatory cytokine IL-10 in serum play leading roles in the formation and development of IBS [42,43]. In addition, abdominal pain and discomfort have been suggested to correlate positively with serum IL-6 and negatively with serum IL-10 [44]. Our data also suggested that FMT intervention obviously increased the level of IL-10 and decreased the levels of IL-6, IL-8, IL-1β, and IFN-γ in chronic diarrhea monkey serum during the intervention, especially on Day-8 (Figure 2B–F). Therefore, FMT can relieve chronic diarrhea and help regulate the immune response.

The intestinal microbiota is linked to changes in gastrointestinal function [43]. Firmicutes with anti-inflammatory functional properties were found to be depleted in IBD [45]. *Lactobacillus* inhibits the overgrowth of opportunistic pathogens in the gut and has been considered an effective probiotic for preventing and treating intestinal diseases in the clinical setting [46,47,48,49]. In this work, we found the reduction in Firmicutes and *Lactobacillus* could be restored by FMT intervention in chronic diarrhea monkeys (Figure S1B,C). The abundance of the *Lactobacillus* species *L. fermentum* and *L. ruminis* CAG_367 also increased during the FMT intervention, especially on Day-8 (Figure 3C). *L. fermentum* is one of the primary SCFA-producing bacterial strains [50] and can also induce the growth of other *Lactobacillus* species [51]. Children with severe acute malnutrition given probiotic supplementation treatment showed enhanced compositions of Lactobacillaceae (*L. ruminis*), reaching similar β-and α-diversities as healthy individuals during follow-up [52]. Erysipelotrichaceae has been implicated in inflammation-related disorders of the gastrointestinal tract [53]. *Erysipelotrichaceae bacterium* 21_3, a sulfur-metabolizing bacteria, is an important microbe in the CRC microbiome [54]. Our results indicated that the abundance of *Erysipelotrichaceae bacterium* 5_2_54FAA and *Erysipelotrichaceae bacterium* 21_3 decreased on Day-8 after FMT intervention. Furthermore, we observed *C. porcorum* (mucin-degrading bacteria [55]) and *D. desulfuricans* to be significantly reduced on Day-8 after FMT intervention (Figure 3C). Mucin degradation may expose intestinal epithelial cells to pathogens, leading to diarrhea [20,56]. *D. desulfuricans* was more abundant in colitis-FMT-recipient mice, which elicited more inflammatory cytokine responses [57].

Shotgun metagenome sequencing is able to capture strain-level differences and functional characterizations. A combination of both can describe the microbiota status better, which is essential for designing effective microbiome-based treatments [58,59]. Using Spearman correlation analysis, we found that seven KEGG pathways were inversely correlated with BSS, indicating that these beneficial metabolic pathways promoted symptom relief. FMT monkeys had increased alanine, aspartate, and glutamate metabolism on Day-8 (Figure 4A). Glutamine is a beneficial amino acid that can be used by the intestinal endothelium, playing a vital role in gut integrity [60,61]. The gut microbiota metabolism of glutamate was shown to be related to human health because there is a decrease in this process in obesity, insulin resistance, and neurological disorders [62]. Additionally, *L. fermentum* contributes to increases in beneficial metabolic pathways. Furthermore, biosynthesis of ansamycins, which was enriched in pre-FMT monkeys, was directly correlated with IL-1β (Figure 4A). Ansamycins are related to antibiotic production, suggesting that colonizers at this time may produce antibiotics to target those who compete with them. Specifically, our analysis revealed the presence of several potential pathogen colonizers, including *D. desulfuricans*, *C. porcorum*, and *Erysipelotrichaceae bacterium* 5_2_54FAA (Figure 4A).

All the ARG subtypes were classified into five primary mechanism categories according to the CARD database [63]. The antibiotic inactivation mechanism category mainly comprised aminoglycoside- and β-lactam-resistance genes. In addition, the efflux pump mechanism category mainly contained multidrug resistance genes. From the results in our study, five CARD classes were directly associated with bacteria related to chronic diarrhea and inversely associated with diarrhea-relieving bacteria (Figure 4B). These results suggested that the alleviation of chronic diarrhea by FMT may be related to its regulation of gut bacteria composition and function.

We found Ascomycota and Basidiomycota to be two dominant phyla in monkey guts, which is consistent with human microbiomes [64]. Furthermore, the Ascomycota/Basidiomycota ratio was higher in chronic diarrhea monkeys after FMT intervention (Figure 5C), suggesting this imbalance between Ascomycota and Basidiomycota may be correlated with chronic diarrhea in monkeys. Moreover, unlike in humans, where *Saccharomyces* and *Candida* are the top two dominant genera, *Trichosporon* and *Cephaliophora* were the two predominant taxa in monkeys, after an unknown genera of high abundance [64]. *Trichosporon spp*. are commonly found commensals of the human skin and gastrointestinal tract [65]. Of *Trichosporon* genus species, the most commonly identified pathogen is *T. asahii*, which reportedly causes more than 70% mortality in invasive trichosporonosis [66]. In this study, the fungal genus *Trichosporon* and fungal species *T. asahii* were found to be decreased in chronic diarrhea monkeys after FMT intervention (Figure 5D,E), suggesting the potential adverse role of *Trichosporon* (*T. asahii*). Additionally, the increase in *Aspergillus* in FMT monkeys suggests a beneficial role for these fungi in relieving diarrhea (Figure 5D). Another study also discovered that *Aspergillus* were reduced in CDI patients compared with healthy controls [64].

Methanogen predominance Archaeal enterotype, which is characterized by a high abundance of Methanobacteriales order, *Methanobrevibacter* genus and *M. smithii* species, has been reported in healthy individuals [28]. Our data indicated there was a *Methanobrevibacter-predominant* enterotype in healthy monkeys (Figure 6B). Lecours et al. reported a higher abundance of *M. stastmanae* in IBD patients (47%) than healthy fecal samples (20%) [67]. Although there was no significant change in the abundance of *M. stastmanae*, it was inversely correlated with anti-inflammatory IL-4 after FMT (Figure 6D). In general, chronic diarrhea monkeys exhibited enteric fungal and Archaeal dysbiosis, and fungal and Archaeal metastasis were correlated with FMT outcomes.

## 5. Conclusions

Human-derived FMT played a certain role in improving the symptoms of chronic diarrhea in monkeys, and the absence of adverse events during the trial period mean that FMT could be an alternative therapy for chronic diarrhea. This notion is supported by our mechanism research that showed FMT helps to regulate the immune response. In addition, there were clear compositional and functional changes in the gut bacteria that were accompanied by alterations in fecal fungi and Archaea after FMT (Figure S2). The current results may help to further the development and application of FMT for chronic diarrhea. However, this study had some limitations. Because of the small scale of the research project and the specificity of the monkey models, we only conducted non-invasive mechanistic studies and there was a lack of conclusions on causality. We propose that further research is needed with a greater sample size to verify causality and identify the precise mechanisms involved.

## Data Availability

The data that support the findings of this study have been deposited into the CNGB Sequence Archive (CNSA) of China National GeneBank DataBase (CNGBdb) with accession number CNP0003519.

## Supplementary Materials

The following supporting information can be downloaded at: https://www.mdpi.com/article/10.3390/biomedicines10123016/s1, Figure S1: Effects of the FMT treatment on the incidence of diarrhea and gut microbiome; Figure S2: Summary of the study findings. Table S1: Summary information of cynomolgus monkeys; Table S2: Bristol stool scale, Table S3: PERMANOVA in microbial species composition.
