# Supplementary material for: Fecal Microbiota Transplantation Could Improve Chronic Diarrhea in Cynomolgus Monkey by Alleviating Inflammation and Modulating Gut Microbiota
Source: Biomedicines. 2022 Nov 23;10(12):3016. doi: 10.3390/biomedicines10123016 (PMC9775123; doi:10.3390/biomedicines10123016)
Supplement: Supplementary file 1 [file biomedicines-10-03016-s001.zip › biomedicines-1996738-supplementary.pdf]

Supplementary Materials

Table S1. Summary information of cynomolgus monkeys

**Sample ID**	**Gender**	**Age （years old）**	**Group**
HC_1	Male	7	HC
HC_2	Male	7	HC
HC_3	Male	7	HC
HC_4	Male	7	HC
0_Diarrhea_1 (0_174317)	Male	5	Day_0
0_Diarrhea_2 (0_180552)	Male	4	Day_0
0_Diarrhea_3 (0_186850)	Male	4	Day_0
2_ Diarrhea_1 (2_174317)	Male	5	Day_2
2_ Diarrhea_2 (2_180552)	Male	4	Day_2
2_ Diarrhea_3 (2_186850)	Male	4	Day_2
4_ Diarrhea_1 (4_174317)	Male	5	Day_4
4_ Diarrhea_2 (4_180552)	Male	4	Day_4
4_ Diarrhea_3 (4_186850)	Male	4	Day_4
8_ Diarrhea_1 (8_174317)	Male	5	Day_8
8_ Diarrhea_2 (8_180552)	Male	4	Day_8
8_ Diarrhea_3 (8_186850)	Male	4	Day_8

Table S2. Bristol stool scale

Typing	Form	Description
1	Nut-like stool	Hard, small pieces, like rabbit dung
2	Dry hard stool	Hard texture with multiple small pieces stuck together, sausage-like
3	Wrinkled stool	The surface is covered with cracks and is sausage-like
4	Banana-shaped stool	Soft texture, smooth surface, sausage-like
5	Soft stool	Soft semi-solid with uneven edges
6	Slightly shaped stool	Soft flakes, porridge with rough edges or no fixed shape
7	Watery stool	Watery, completely liquid without solids

Table S3. PERMANOVA in microbial species composition

Phenotypes	F	R^2	P value
Grouping	1.388523	0.335512	0.102897
BSS	2.137205	0.13244	0.043956
IL-6	2.028222	0.126541	0.040959
IL-8	0.901766	0.060514	0.484515
IL-1β	1.268482	0.083078	0.236763
IFN-γ	1.053468	0.069982	0.390609
IL-4	0.629051	0.043	0.811189
IL-10	0.653095	0.04457	0.778222

F represents the F-statistic: alarger F value indicates that the between-group variation is greater than within-group variation. R^2 represents the variation explained by the model. q represents the q-value calculated from permutation.

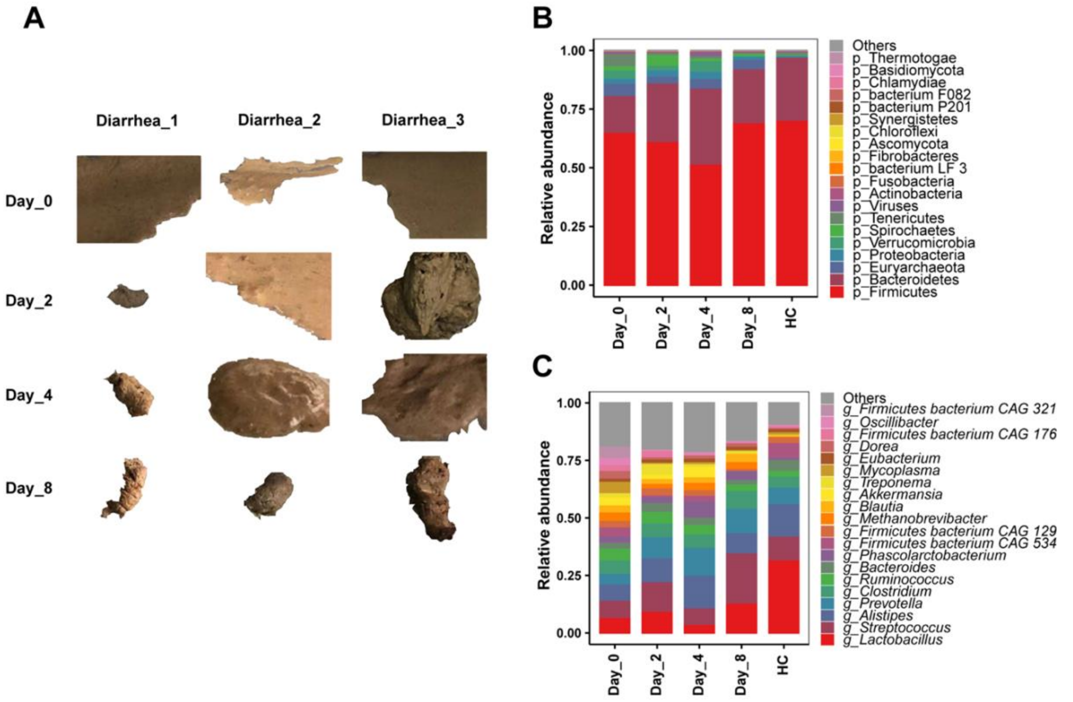

Figure S1. Effects of the FMT treatment on the incidence of diarrhea and gut microbiome. (**A**) Images of the fecal samples collected from Day_0 (n = 3), Day_2 (n = 3), Day_4 (n = 3) and Day_8 (n = 3). (**B**) The distribution of dominant bacterial phyla in all 5 groups. (C) The distribution of dominant bacterial genus in all 5 groups. HC, asymptomatic monkeys (n = 4); Day_0, chronic diarrhea monkeys before FMT intervention (n = 3); Day_2, Day_4, Day_8, chronic diarrhea monkeys after FMT intervention (n = 3 respectively).

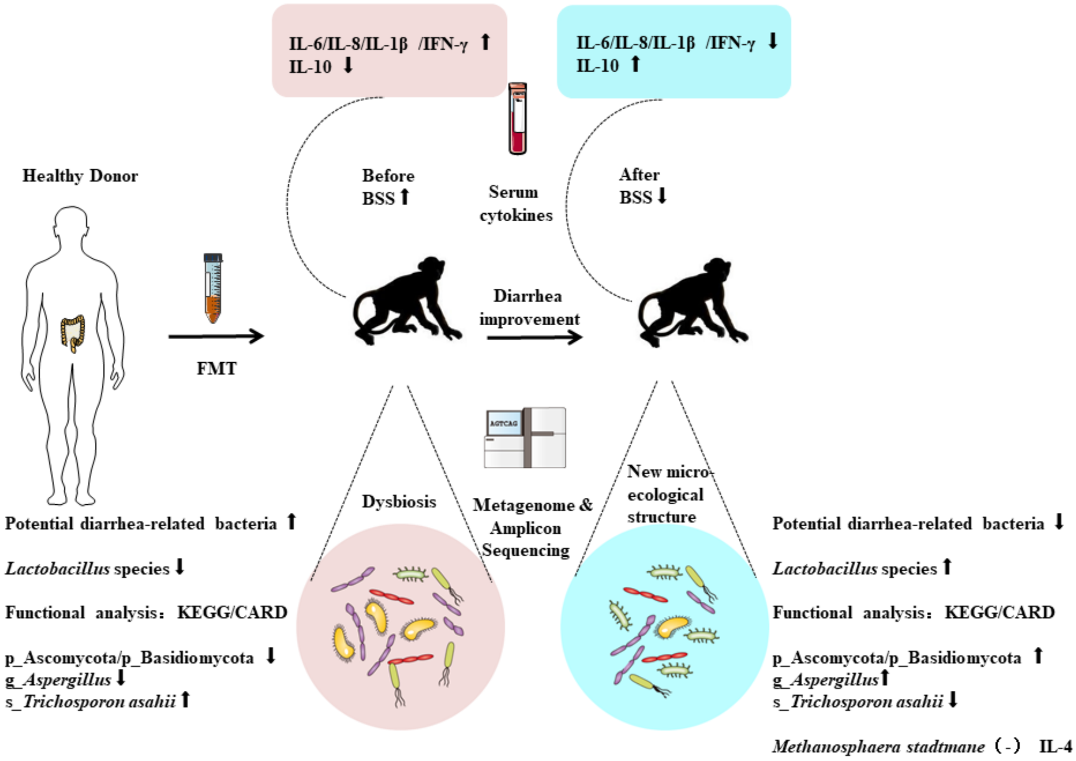

Figure S2. Summary of the study findings.
